# Characteristics and long-term outcomes of perineal endometriosis: A retrospective study

**DOI:** 10.1097/MD.0000000000020638

**Published:** 2020-06-05

**Authors:** Yu Liu, Ruyu Pi, Hong Luo, Wei Wang, Xia Zhao, Xiaorong Qi

**Affiliations:** a Department of Gynecology and Obstetrics, Key Laboratory of Obstetrics & Gynecologic and Pediatric Diseases and Birth Defects of Ministry of Education; b Department of Diagnostic Ultrasound; c Department of Pathology, West China Second University Hospital, Sichuan University, Chengdu, China.

**Keywords:** endometriosis, episiotomy, extra-pelvic, gonadotropins releasing hormone agonist, perineum, vulva

## Abstract

To summarize the clinical features, diagnosis, and treatments of perineal endometriosis (PEM).

We retrospectively studied the clinical data of 35 patients with PEM between April 2012 and December 2018 in West China Second Hospital. Patients were divided into the gonadotropins releasing hormone (GnRH) agonist group and non-GnRH agonist group.

The main clinical symptom was vulvar painful swellings related to menstrual cycles. Thirty-three patients’ lesions (94.29%) were on the episiotomy scar while 1 case was at the opposite side of the scar. We even found 1 nullipara was diagnosed as PEM. Ten patients (28.57%) were found with anal sphincter involvement. All patients received complete excision of PEM. The recurrence rate of GnRH agonist group was 7.69% (1/13), while the rate of non-GnRH agonist group was 18.75% (3/16).

Most PEM was associated with episiotomy history, but PEM could also exist in nullipara. Complete excision of PEM was inevitable. The effect of GnRH agonist on recurrence of PEM needs further studies.

## 1 Introduction

Endometriosis is a benign, inflammatory disease and could cause chronic pelvic pain, dysmenorrhea, and infertility. It is estimated that endometriosis affects about 6% to 10% women of reproductive age.^[1,2]^ Endometriosis occurs in pelvic, as well as out of pelvic, such as umbilicus, abdominal scar, the gastrointestinal tract, the urinary system, vagina, or perineum.

Perineal endometriosis (PEM) is a rare subtype of extra-pelvic endometriosis, taking up 0.17% to 0.37% among endometriosis, often associated with a history of episiotomy.^[3–5]^ Ectopic endometrial tissues are hormone-responsive tissues that bleed or enlarge in the menstrual cycles. The main clinical symptom of PEM is painful and enlarged nodules at perineum associated with menstruation. When perineal lesions invade the sphincteric muscular area, it is called PEM with anal sphincter involvement.^[3,4]^ PEM with anal sphincter involvement has risk of causing fecal incontinence episodes and fistula in surgery. Currently, there is no exact treatment guideline for PEM. And all patients with PEM experienced surgical treatments to remove the lesions at perineum, with or without medical treatments.^[4,6–9]^ Medical treatments include nonsteroidal anti-inflammatory drugs, oral contraceptives, gonadotropins releasing hormone agonist (GnRH agonist) and antagonists, and danazol. These treatments are with primary goal of managing pain and associated symptoms and reducing recurrence. Although some local recurrences were reported after lesions excision surgery, the accurate recurrence rate of PEM was currently unclear. Majority of the studies about the management and treatments of PEM was derived from case reports, and the treatments and management of PEM are debatable.

In this retrospective study, we aimed to summarize the clinical features of PEM and discuss prior treatments for it, as well as the recurrence rate of patients whether used GnRH agonist postoperatively or not.

## 2 Materials and methods

We collected 35 cases diagnosed as PEM and treated surgically at West China Second Hospital from April 2012 to December 2018. We retrospectively analysis their medical records, including medical history, physical examination, ultrasound examination, surgical records, and medical treatment. The study was approved by the ethics committee of West China Second Hospital. Written informed consent was not obtained, as this was a retrospective study and no identifying information of individual patient.

All patients were evaluated with pelvic ultrasonography. All patients received complete resection of PEM lesions and primary sphincteroplasty if necessary. Complete resection was defined as a resection 0.3 to 0.6 cm outside the edge of PEM and a clear margin confirmed by the pathology. All patients were followed up in outpatient clinic after surgery. We also evaluated the latent period and PEM recurrence. The latent period was defined as the time since the latest delivery to when patients presented symptoms such as perineal pain or nodule. For the patient without any parity history, latent period was not recorded. PEM recurrence was defined as the lesion or symptoms occurred again in the perineum after treatment.

A telephone interview was conducted to patients in June 2019. Only 1 patient (2.86%) was lost to follow-up. The follow-up lasted 7 to 86 months (the median was 49 months).

To determine the effect of GnRH agonist on recurrence of PEM after surgery, we compared the recurrence rate between patients who used GnRH agonist after surgery and patients who not used. The patients treated with GnRH agonist before surgery were excluded to eliminate interference. According to patients whether used GnRH agonist after surgery or not, we defined as the GnRH agonist group and non-GnRH agonist group, respectively. The GnRH agonist group included 13 patients, while non-GnRH agonist group included 16 patients. Five patients were excluded because of preoperative GnRH agonists treatment.

## 3 Results

The mean ages of 35 patients at the time of surgery was 33.44 (range, 25–48) years. Thirty-four patients (97.14%) had a history of vaginal delivery, while 1 patient (2.86%) had no pregnancy before. The average gravidity was 2.51 and parity was 1.11. The mean latent period of these 34 cases was 42.44 months (range, 1–120). All patients presented vulvar swellings enlarged during menstrual cycles and menstruation-related pain. The clinical characteristics and treatment details for the 35 patients are listed in the Table 1.

**Table 1:**
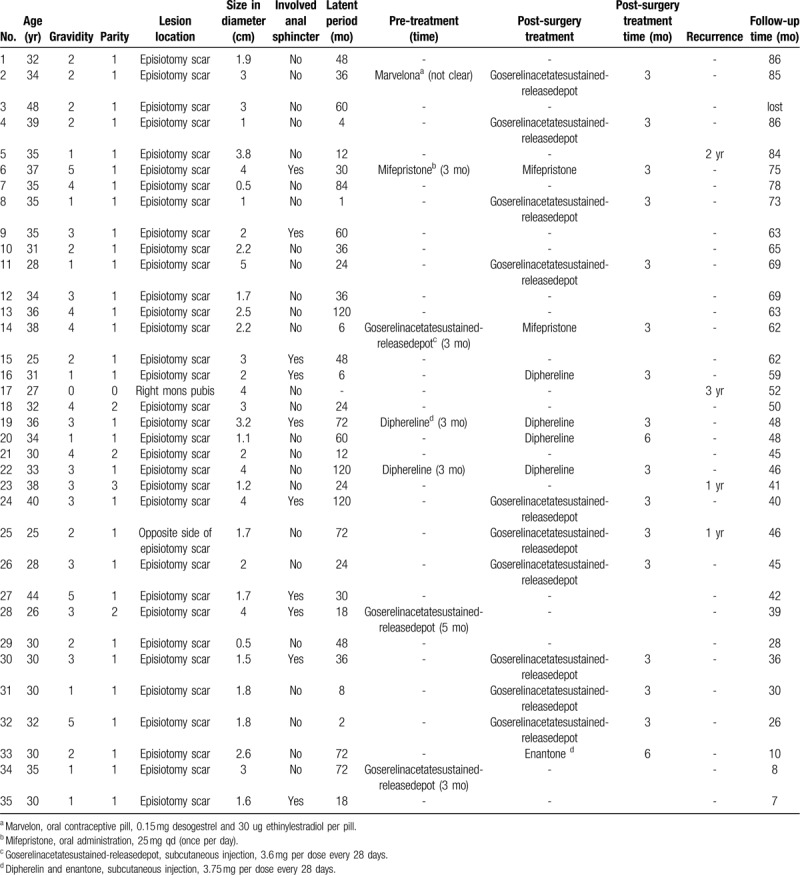
The clinical characteristics and treatments for 35 patients of perineal endometriosis.

As for the lesion location, 33 patients (94.29%) were on the episiotomy scar in our study. PEM also was found at the opposite side of the scar in 1 case, as well as at right mons pubis in 1 case without history of reproduction or pregnancy. The size of PEM nodules differed among patients, ranging from 0.5 to 5 cm in diameter with a mean value of 2.39 cm. Ten patients (28.57%) were found with anal sphincter involvement.

**Figure 1:**
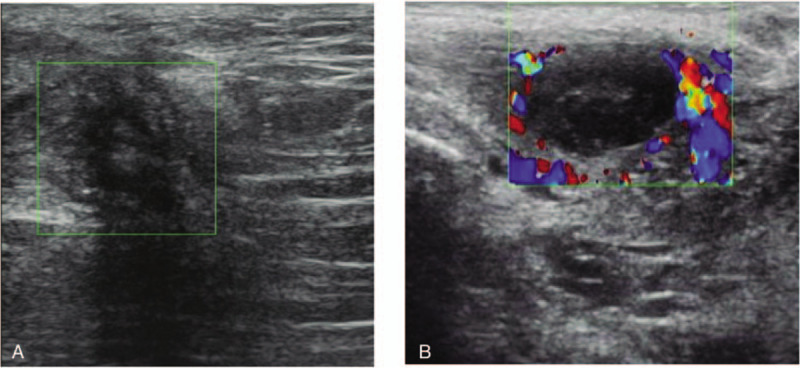
The typical ultrasonographic images of perineal endometriosis patients. The presentation of perineal endometriosis on ultrasound examination could be irregular hypoechoic without blood flow signal around (A), or a cyst with clear border and vascular signals around (B).

Serum CA125 slightly increased in 4 patients among 11 patients who underwent CA125 test. Fifteen patients (42.86%) had perineal subcutaneous ultrasonography before surgery. The reports showed irregular hypoechoic or cystic mass at the perineum, with blood flow signals around the mass or not (Fig. 1). Ultrasound was performed on all these women. Five women had coexistent pelvic endometriosis.

**Figure 2:**
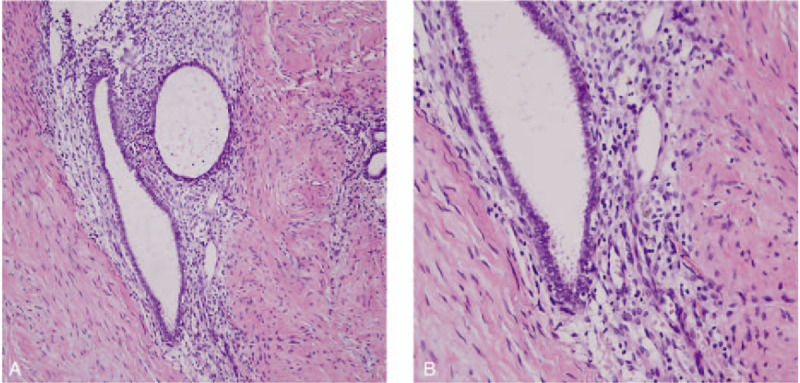
The pathology of perineal endometriosis lesions. Pathologic examination of excised lesion at perineum showed endometrial glands and stroma infiltrated into the muscle under microscope: 100X(A), 200X(B).

All patients received surgical treatment with complete excision of PEM lesion, with a resection margin of 0.3 to 0.6 cm beyond the edge of PEM. Primary anal sphincteroplasty was conducted if necessary. Pathologic examination confirmed the diagnosis as endometrial glands and stroma infiltrated into the muscle under microscope (Fig. 2). All patients recovered without surgical complications.

The medical treatment for PEM includes oral contraception (marvelon), anti-progestins (mifepristone) and GnRH agonist (including goserelin acetate sustained-release depot, dipherelin, and enantone). The usages and dosage of each medicine were listed in the Table 1. Seven patients received preoperative treatment in order to reduce the size of PEM lesions and relief syndromes (5 patients received GnRH agonist for 1–5 months, 1 patient received marvelon, and 1 patient received mifepristone). As for the postoperative treatment, 18 patients (51.43%) were untreated, while 2 patients (5.71%) received mifepristone and 15 patients (42.86%) were treated with GnRH agonist. The duration of postoperative treatment was 3 or 6 months. The recurrence rate was 7.69% (1 of 13 patients) in the GnRH agonist group, compared with 18.75% (3 of 16 patients) in the non-GnRH agonist group.

## 4 Discussion

PEM is a rare type of endometriosis with typical clinical characteristics including enlarged perineal nodules, menstruation-related pain, and vaginal delivery history with episiotomy. Consistent with other reports, most of PEM patients had a history of vaginal delivery and episiotomy.^[4,6,10]^ Patients could be diagnosed as PEM according to typical clinical characteristics and imaging examination features. The definitive diagnosis relies on pathology of lesions. The fine needle aspiration cytology may be considered for preoperative diagnose of PEM.^[11]^ Although most of PEM patients have vaginal delivery and episiotomy history, nulliparous women also have possibility being diagnosed as PEM. In our study, a patient with no gravidity history was diagnosed as PEM. If nullipara has painful nodules at perineum related to menstrual cycle, PEM should be considered. Consisted with Li J et al reported, CA125 slightly increased in some patients, but was not effective in diagnosis of PEM.^[5]^

The mechanism of PEM is currently unclear. It was supposed to be associated with episiotomy or perineal injury. During vaginal delivery, endometrium could implant into the perineum episiotomy scar and then developed to PEM lesion, that is, the implanted theory.^[12]^ Retrograde menstruation hypothesis and the metastatic theory could also explain the development of PEM. Retrograde fragments of menstrual endometrium pass through the fallopian tubes, then implant and persist on peritoneal surfaces.^[13]^ And the metastatic theory that retrograde menstruation metastases through peritoneum, lymphatic duct to the distant locations, such as lung, gastrointestinal tract, perineum and vagina, could explain some cases of PEM. PEM could also be found at the opposite side of episiotomy scar, or even in nulliparous women. And the nulliparous patient of our study, she presented painful nodule at right mons pubis, closed to the inguinal region, where round ligament and occasionally the canal of Nuck pass through. Wolfhagen et al and Mazzeo et al have reported inguinal endometriosis and vulva endometriosis in canal of Nuck, a peritoneal diverticulum.^[14,15]^ The PEM lesion near the inguinal region may be caused by retrograde menstruation or hematogenous endometriosis tissue migrated through the ligament.

Patients diagnosed as PEM are suggested to remove the lesion in surgery, and completely excision is critical for reducing local relapse.^[4,5]^ Endometriosis at perineum may invade neighbor structure, especially anal sphincter. Anal sphincter involvement is associated with the treatment of PEM. Patients diagnosed as PEM with anal sphincter involvement have risks of dyspareunia, fecal incontinence episodes and fistula.^[4]^ Preoperative examinations, including ultrasound and MRI, could indicate the relation between PEM nodule and anal sphincter.

PEM patients treated with GnRH-agonist after surgery showed lower recurrence rate compared with those in non-GnRH agonist group (6.67% vs 18.75%). However, considering sample size and compounding variables may influence the outcome, statistical analysis could not be down. GnRH-agonists lead to down-regulation of the pituitary GnRH receptor with a subsequent decrease in pituitary secretion of LH and FSH, which results low circulating estradiol and progesterone.^[16]^ In our study, the post-surgical usage of GnRH agonist showed a potential decrease of recurrent risk for patients with PEM. It is consistent with a report that postoperative GnRH agonists could reduce the relapse of endometriosis in perineum^[8]^. A meta-analysis of postoperative GnRH agonist treatment in endometriosis suggested that only long-term treatment (6 months) could prevent the recurrence of endometriosis, rather than 3 months duration treatment.^[17]^ In our study, the duration of GnRH agonists was 3 months in most cases (14/15), only 1 case used Dipherelin for 6 months. However, it is still unclear whether prolong the duration of GnRH agonists treatment for PEM to 6 months or even longer could reduce the recurrence. Prospective studies with large samples are needed.

One limitation of this study was the limited sample. Since PEM is rare extra-pelvic endometriosis, the number of PEM cases in a single center was limited. And in our study, only cases with surgical treatment were collected. Those PEM patients who only received medicine therapy were excluded and their prognosis was unknown. Multicenter cooperation explore more in further study.

In conclusion, most PEM was associated with episiotomy history, but PEM could also exist in nullipara. The typical symptoms were vulvar swellings enlarged during menstrual cycles with menstrual pain. Complete excision of PEM was inevitable. Postoperative GnRH agonist treatment may reduce the recurrent risk of PEM. Further studies with large sample sizes are needed to confirm the therapeutic benefit.

## Author contributions

All authors (YL, RP, HL, WW, XZ, XQ) read and approved the final manuscript. YL and RP wrote the initial manuscript. HL and WW offered ultrasonic and pathological pictures. XZ and XQ contributed new ideas and revised the manuscript and approved the final version.

**Conceptualization:** Xiaorong Qi.

**Data curation:** Yu Liu, Ruyu Pi, Hong Luo, Wei Wang.

**Writing – original draft:** Yu Liu, Ruyu Pi.

**Writing – review & editing:** Xia Zhao, Xiaorong Qi.
